# Lipidomics-Assisted GWAS (lGWAS) Approach for Improving High-Temperature Stress Tolerance of Crops

**DOI:** 10.3390/ijms23169389

**Published:** 2022-08-20

**Authors:** Velumani Pranneshraj, Manjeet Kaur Sangha, Ivica Djalovic, Jegor Miladinovic, Maduraimuthu Djanaguiraman

**Affiliations:** 1 Department of Biochemistry, Punjab Agricultural University, Ludhiana 141004, India; 2 Institute of Field and Vegetable Crops, National Institute of the Republic of Serbia, Maxim Gorki 30, 21000 Novi Sad, Serbia; 3 Department of Crop Physiology, Tamil Nadu Agricultural University, Coimbatore 641003, India

**Keywords:** high temperature, tolerance mechanisms, membrane lipids, lipidomics, photosynthesis, genotype, phenotype, GWAS, breeding

## Abstract

High-temperature stress (HT) over crop productivity is an important environmental factor demanding more attention as recent global warming trends are alarming and pose a potential threat to crop production. According to the Sixth IPCC report, future years will have longer warm seasons and frequent heat waves. Thus, the need arises to develop HT-tolerant genotypes that can be used to breed high-yielding crops. Several physiological, biochemical, and molecular alterations are orchestrated in providing HT tolerance to a genotype. One mechanism to counter HT is overcoming high-temperature-induced membrane superfluidity and structural disorganizations. Several HT lipidomic studies on different genotypes have indicated the potential involvement of membrane lipid remodelling in providing HT tolerance. Advances in high-throughput analytical techniques such as tandem mass spectrometry have paved the way for large-scale identification and quantification of the enormously diverse lipid molecules in a single run. Physiological trait-based breeding has been employed so far to identify and select HT tolerant genotypes but has several disadvantages, such as the genotype-phenotype gap affecting the efficiency of identifying the underlying genetic association. Tolerant genotypes maintain a high photosynthetic rate, stable membranes, and membrane-associated mechanisms. In this context, studying the HT-induced membrane lipid remodelling, resultant of several up-/down-regulations of genes and post-translational modifications, will aid in identifying potential lipid biomarkers for HT tolerance/susceptibility. The identified lipid biomarkers (LIPIDOTYPE) can thus be considered an intermediate phenotype, bridging the gap between genotype–phenotype (genotype–LIPIDOTYPE–phenotype). Recent works integrating metabolomics with quantitative genetic studies such as GWAS (mGWAS) have provided close associations between genotype, metabolites, and stress-tolerant phenotypes. This review has been sculpted to provide a potential workflow that combines MS-based lipidomics and the robust GWAS (lipidomics assisted GWAS-lGWAS) to identify membrane lipid remodelling related genes and associations which can be used to develop HS tolerant genotypes with enhanced membrane thermostability (MTS) and heat stable photosynthesis (HP).

## 1. Introduction

Across the globe, crop yield is affected by various abiotic stresses like high temperatures (HT), freezing, drought, salinity, heavy-metal toxicity, etc. HT is the foremost among the abiotic stresses because of increasing greenhouse gas emissions. The sixth IPCC Assessment report has highlighted that all regions of the world will be warmer, and a 1.5 °C rise in global temperature will create increasing high-temperature waves, longer warm seasons, and shorter cold seasons—which could be more severe when the rise is 2 °C [1].

Plants are continuously exposed to stressful environments because of their sessile nature. To some extent, the plants can withstand HT stress through physiological changes and frequently altering their metabolisms. In particular, the plants respond by producing compatible solutes that can organise proteins and cellular structures involved in osmotic adjustment and redox homeostasis [2,3]. The membrane lipid metabolism is also altered in response to HT stress. An increase in membrane fluidity is one of the immediate consequences of HT stress. Plants coordinate the lipid metabolism to maintain the structure and integrity of membranes [4]. High-temperature tolerant genotypes of wheat (*Triticum aestivum* L.) and soybean (*Glycine max* L.) increased the saturated lipid content to avoid the temperature-induced hyper fluidity of membranes. In this context, lipidomics, a branch of metabolomics, would be a valuable tool in evaluating the effect of high-temperature stress on plants. Lipidomics is the comprehensive study of all lipid species in a biological system, and assessing alterations in the lipid contents and compositions in response to environmental conditions will be a valuable tool in developing stress-tolerant lines [5].

Lipid compositional variation in response to HT stress has been recorded in several plants such as *Arabidopsis thaliana* [6], wheat [7,8], soybean [9], and Ethiopian mustard (*Brassica carinata*) [10]. Djanaguiraman et al. [7] have reported that in wheat, the decrease in photosynthetic rate under HT stress is due to thylakoid membrane damage, lipid composition alterations, and oxidative damage of organelles. The membrane lipid alterations responsible for membrane stability under HT stress are orchestrated by the coordinated activities of lipid desaturating, oxidising, glycosylating, and acylating enzymes [7,11]. The study also indicated that certain oxidized lipid species could be biomarkers for screening HT stress-tolerant genotypes.

The HT stress damage is attributed to hyper-fluidity of membrane lipids. Membrane organization is disrupted, and the lipid composition is altered. The thylakoid membrane is highly susceptible to HT stress. High-temperature stress damages the photosynthetic light reactions of the thylakoid membrane and decreases the quantum yield of PSII [8]. The frequency of non-bilayer forming lipids in membranes is increased. Lipid–protein interaction is disrupted due to the denaturation of lipids and proteins. High-temperature stress also increases the production of reactive oxygen species (ROS), which causes membrane lipid peroxidation and accumulation of cytotoxic carbonyl groups in leaves. Thus, maintaining the integrity and fluidity of the membrane and preventing lipid peroxidation are of fundamental importance for plants to survive under HT [12,13,14]. 

Mutation studies have reported that several gene mutations related to membrane lipid synthesis, transport, and desaturation are associated with HT stress tolerance or susceptibility [15,16,17]. Hence, uncovering the molecular mechanisms responsible for tolerance resulting from the membrane lipid alterations becomes essential. An integrated approach utilising metabolic and transcriptome data has been used to discover genes related to that metabolism. However, the genetic determinants underlying the quantitative variation in the metabolites or lipids responsible for the desired trait can be determined better with a robust integrated multi-omics approach such as a metabolome-assisted genome-wide association study (mGWAS). This review will focus on the idea of utilising a forward genetic analysis integrating the lipidome and quantitative genetics to perform lGWAS (Lipidomics-assisted Genome-Wide Association Study) (Figure 5) which could reveal the genetic associations for the intermediate phenotype–LIPIDOTYPE of plants responsible for membrane thermostability (MTS) and high-temperature stable photosynthesis (HP) under HT, which can be further characterised and utilised in breeding for HT stress-tolerant genotypes.

## 2. Diversity of Plant Lipids and Study of Lipids

Lipids are not generally derived directly from the central dogma flow, i.e., they are not genetically encoded like proteins [18]. Instead, they are produced and metabolised by multiple enzymes, which are influenced by various factors. A steady state of the cellular lipidome cannot be maintained, i.e., enzymes and environmental cues constantly modify the lipids. Lipids have several primary cellular functions, such as being the major components of biological membranes responsible for maintaining the structure and integrity of membranes and the cell ultrastructures [19], acting as an energy source [20], signalling molecules [21], and as substrates for post-translational protein–lipid modifications. In plants, lipids play significant roles in photosynthesis, signal transduction under stress conditions, vesicle trafficking and secretion, and cytoskeletal rearrangements [22,23]. Lipids exhibit extreme combinational and structural diversity. It is estimated that the total number of lipids in a cell or tissue varies in the thousands, with various unique lipid species in variable abundance [24]. The diversity of lipids can be defined in two ways: (1) chemical diversity—chemically diverse structures such as stereo-isomers, and various head groups, and (2) compositional diversity—ratio of different lipids present in different species, tissues/cells in an organism, in different organelles, and in different membrane leaflets and even sub-domains. In other words, lipids are unevenly distributed between the different membranes of the cell [25]. Further, lipids become more diverse due to multiple combinations of a variety of head groups and tail fatty acids, with fatty acids further varying in chain length and saturation levels (number of double bonds) [24]. Several lipid modifications can also contribute to the diversity of membrane lipids, such as acylation, glycosylation, fatty acid desaturation and oxidation, which happens under specific conditions [8].

Plant membrane lipids fall into three broad classes: glycerolipids (phospholipids, galactolipids, sulfolipids, triacylglycerols), sphingolipids, and sterols, with each lipid class varying greatly in their properties [26]. Lipids are characterised by their varying backbone structure, variable hydrophilic head groups, and the attached variable-length hydrophobic fatty acid chains [27]. Phospholipids containing phosphorous in head groups are the major constituents of cell membranes except for chloroplast, where galactolipids dominate. The simplest phospholipid is phosphatidic acid (PA), a precursor for various other lipids [28]. Different head groups of phospholipids include glycerol, choline, ethanolamine, serine, and inositol, which are referred as phosphatidylglycerol (PG), phosphatidylcholine (PC), phosphatidylethanolamine (PE), phosphatidylserine (PS), and phosphatidylinositol (PI), respectively [25]. The photosynthetic thylakoid membranes of the chloroplast are dominantly made of non-phosphorous galactolipids (monogalactosyldiacylglycerol; MGDG and digalactosyldiacylglycerol; DGDG) and sulfolipids (sulfoquinovosyldiacylglycerol; SQDG), and one phosphorous-containing lipid phosphatidyl glycerol (PG) to some extent, but proven to be highly indispensable for PSII functioning [29,30]. The significance of the galactolipid predominance of nearly 80% in chloroplast membranes is a possible evolutionary strategy of plants to maintain photosynthesis even under phosphate-limiting conditions [31]. Plant sphingolipids reach up to 10% of the total lipids in plants, depending on the species and tissues, and it is made of a ceramide backbone with a long chain base and a long-chain fatty acid esterified to it. They are grouped into four classes: glucosylceramides (GCer), glycosylinositol phosphoceramides (GIPC), ceramides (Cer), and free long-chain bases (LCB) [32,33]. Sterols are ubiquitous components of the eukaryotic cells reaching up to 10% in membranes [34], with plant sterols or phytosterols differing from one another in the 24th carbon atom of the side chain by either methyl or an ethyl group [35]. About 250 phytosterols have been identified in plants, such as campesterol (24-methyl sterols), stigmasterol and β-sitosterol (24-ethyl sterols) present in various forms such as free sterols, sterol esters, steryl glycosides (SG) and acylated steryl glycosides (AcSG) [35,36]. Phytosterols also act similarly to cholesterol in mammalian cells, regulating membrane fluidity and permeability [37]. 

Lipidomics, a branch of metabolomics studying lipids, is more than just the complete characterization of all lipids in a system. It is the comprehensive understanding of the influence of all lipids on a biological system concerning signalling, membrane architecture, transcriptional and translational modulation, cell–cell and cell–protein interactions, and response to environmental changes [38]. The vast diversity of lipids necessitates analytical techniques with high separation power. Advances in mass spectrometry-based lipidomics platforms with greater resolution power enable high-throughput lipid analysis at a relatively quick scale [39]. Two analytical lipidomics approaches are available to profile the lipids: targeted and untargeted lipidomics. As the name suggests, the targeted approach focuses on a detailed list of lipid species, mainly of the same lipid class, subclass, or several classes. An untargeted method is employed to screen the entire lipidome of an organism for which no previous information is available [24,40]. 

## 3. Membrane Lipid Biosynthesis

**Figure 1:**
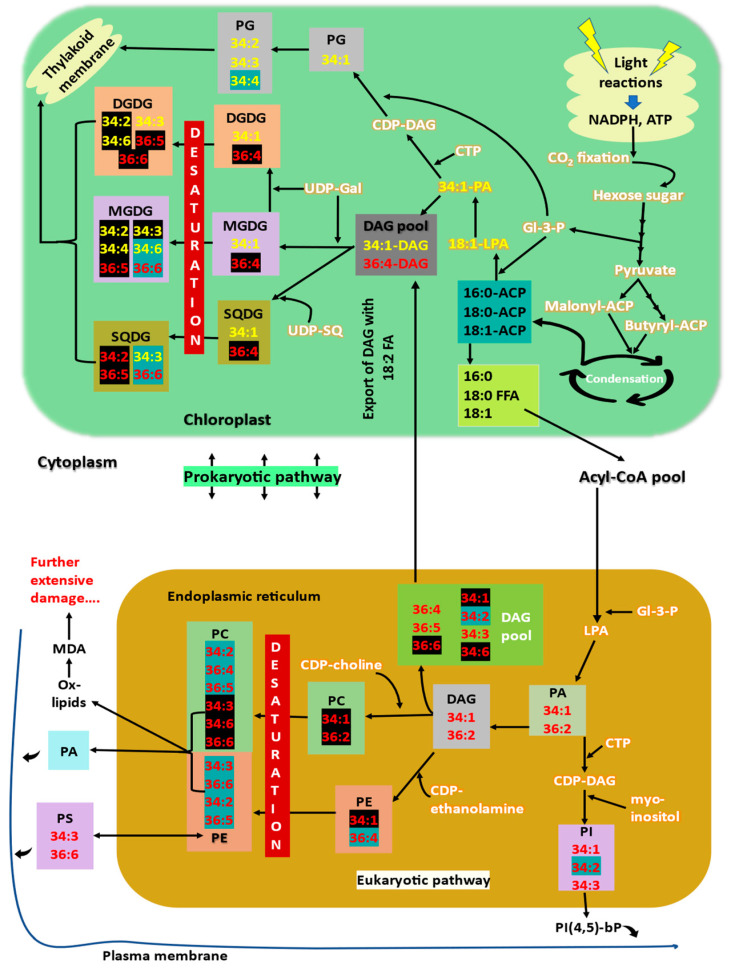
Overview of the fatty acid de novo and lipid biosynthetic pathways (prokaryotic and eukaryotic) in chloroplast and endoplasmic reticulum with major lipid remodelling changes occurring in the pathways under high-temperature conditions. Prokaryotic pathway lipids are represented in yellow and eukaryotic lipids are represented in red. Lipids highlighted in blue are decreased, and those highlighted in black increase under high temperatures. The figure showing the steps of fatty acid and lipid biosynthesis is modified from Holzl and Dormann [46] and Higashi and Saito [63]. Collectively, the light and dark reactions of photosynthesis yield pyruvate. The pyruvate dehydrogenase in chloroplast links the pyruvate metabolism and de novo fatty acid biosynthesis yielding the acetyl-CoA. Several condensation steps occur to provide 16:0-ACP, 18:0-ACP, and 18:1-ACP for prokaryotic lipid synthesis pathway or exported into ER for utilisation in eukaryotic lipid synthesis pathway. The fatty acids exported to ER are then made into DAGs and exported back to chloroplast for plastidic lipid synthesis. Under high temperatures, overall contribution of prokaryotic pathway for galactolipid synthesis is reduced, and eukaryotic pathway is increased. Abbreviations: FFA, free fatty acid;16:0, palmitic acid; 16:3, hexadecatrienoic acid; 18:0, stearic acid; 18:1, oleic acid; 18:2, linoleic acid; 18:3, linolenic acid; ACP, acyl carrier protein; Gro-3-P, glyceraldehyde-3-phosphate; CDP, cytidine diphosphate; CTP, cytidine triphosphate; UDP, uridine diphosphate; DAG, diacyl glycerol; LPA, lysophosphatidic acid, PA, phosphatidic acid; PG, phosphatidyl glycerol; PC, phosphatidyl choline; PE, phosphatidyl ethanolamine; PS, phosphatidyl serine; PI, phosphatidyl inositol; PI(4,5)-bP, phosphatidyl inositol-4,5-bisphosphate; MGDG, monogalactosyl diacylglycerol; DGDG, digalactosyl diacylglycerol; SQDG, sulfoquinovosyl diacylglycerol; MDA, malondialdehyde.

The biosynthesis of lipids can be conceptualized into three phases: (a) assembly of phosphatidic acid (PA), (b) formation of free/activated DAG backbone, and (c) formation and assembly of head groups to make the whole lipid molecule. After completion of step (c), acyl groups attached to the backbone are desaturated by various fatty acid desaturases (FADs) to make the actual lipid molecular species [27]. Two distinct and discrete pathways exist for the membrane lipid synthesis and assembly utilizing the fatty acyl pool: eukaryotic (cooperative between endoplasmic reticulum ER and chloroplast) and prokaryotic (chloroplast) pathways located in the ER and chloroplast, respectively (Figure 1) [41,42,43,44]. Both pathways are compartmentalized by membrane barriers but are closely coordinated to meet the overall demand for cellular membrane biogenesis and maintenance [42]. The relative contribution of the pathways to overall glycerolipid production is flexible and varies depending on several factors [45]. The prokaryotic pathway synthesizes several glycerolipid classes: three galactolipid classes, MGDG, DGDG, and TGDG, and a sulfolipid class, SQDG [46]. The eukaryotic pathway (in ER) synthesizes phospholipid classes such as PC, PE, PI, and PS [27].

Almost 95% of the fatty acids in plant cells are synthesized by type I fatty acid synthases (FAS) in the chloroplasts. The major products of de novo fatty acid synthesis are 16:0-ACP and 18:1-ACP [46]. Fatty acids are formed in the chloroplasts and used in the prokaryotic pathway for lipid synthesis or transported to ER for eukaryotic pathway utilization. The first product of the prokaryotic course is 18:1, 16:0-PA and the eukaryotic pathway is 18:1/16:0, 18:1-PA (*sn*1, *sn*2) (Figure 1) [47,48]. PAs produced by both routes can be dephosphorylated into DAGs by phosphatases [49], which are later made available for the synthesis of plastidic as well as extra-plastidic glycerolipids [50]. DAGs made by eukaryotic pathway in ER are actively transported into chloroplasts via physical contact sites between ER and outer chloroplast envelope to increase the DAG pool for plastidic lipid synthesis [51,52]. The contribution of DAGs by both pathways varies depending on the plant species. The galactolipid MGDG is made by adding galactose to DAG by MGDG synthases, and DGDG is made by adding galactose to MGDG by DGDG synthases [43,53]. Sulfolipid SQDG is created by transferring sulfoquinovose from UDP-sulfoquinovose to DAG by glycosyltransferase [54]. Fatty acids of these galactolipids are then desaturated by several fatty acid desaturases (FADs) [55]. 

Phosphatidyl glycerol (PG) is synthesised in chloroplasts via the prokaryotic pathway, ER, and mitochondria. PG is made by the CDP-DAG pathway in which DAG is transferred from CDP-DAG to glycerol-3-phosphate, followed by dephosphorylation [46,56]. Phosphatidyl ethanolamine (PE) is synthesised by converting serine to ethanolamine, and its further attachment to the DAG backbone or ethanolamine headgroup transferred to DAG from CDP-ethanolamine (Figure 1). Phosphatidylcholine (PC) is formed by the head group activation pathway utilising either choline or PE [57,58]. Phosphatidylserine (PS) is assembled by the base-exchange reaction between the serine and ethanolamine head group of PE [59]. Phosphatidyl inositol (PI), a minor class of phospholipid and an essential precursor for the synthesis of signalling PIPs, is made by the attachment of the inositol headgroup to the DAG backbone [27]. Triacylglycerols (TAG) can be synthesised in either cytosol or chloroplasts. Cytosolic TAGs are assembled in ER through the Kennedy pathway, which transfers the fatty acyl chains from the cytosolic acyl-CoA pool to the glycerol-3-phosphate [60]. An acyl-CoA independent pathway also contributes to the TAG synthesis utilising PC as an acyl donor, transferring fatty acid chain onto a DAG molecule [61]. The level of desaturation of fatty acids in PC determines the desaturation level of DAG precursors for seed TAG and plastidic lipid synthesis. The actual pathway of TAG synthesis in chloroplast has not yet been elucidated [46]. Phytosterols are synthesised in the ER via the first mevalonate pathway to produce squalene and the second squalene cyclization. Steryl glycosides (SG) are made by glucosylation of the sterols by UDP-glucose: sterol glucosyltransferase [62]. Extensive details about membrane lipid biosynthesis have been reviewed by Nakamura [27] and Holzl and Dormann [46].

## 4. Mass Spectrometry-Based Lipid Profiling

The cellular lipidome is so diverse that a simple analytical technique will not sufficiently profile all the lipids in a cell or tissue [24,40,64]. Numerous methods have been developed for the same, and lipidomics is now gaining momentum to emerge as an additional dimension of omics. Available technologies for lipid analysis are mass spectrometry (MS)**,** fluorescence spectroscopy, nuclear magnetic resonance (NMR), Fourier-transform infrared spectroscopy (FT-IR), and UV-Vis spectroscopy [18,65]. Traditional methods for analysing lipids lack sensitivity and resolution, are time-consuming and can study only a selected class or set of cellular lipids [23]. Recent advances in soft ionization techniques, such as Electrospray Ionisation-Mass Spectrometry (ESI-MS), have revolutionized lipidome analysis [66]. Mass-spectrometry-based techniques are of prime focus in this review. A typical MS system has three components: (1) an ion source to generate gaseous ions of the substance of interest, (2) an analyser to resolve the generated ions into their characterised mass components based on their *m*/*z* ratio, and (3) the detector, to detect the ions and record the relative abundance of each resolved ion species. Several ion sources can be coupled with mass analysers [67]. Ion sources such as electrospray ionisation (ESI), atmospheric pressure chemical ionisation (APCI), matrix-assisted laser desorption ionisation (MALDI), desorption ionisation (DESI), and direct analysis in real-time (DART) are available. ESI [68,69] and APCI [70] are commonly used ionisation method for lipid studies. Mass analysers used in MS include triple quadrupole (QqQ), ion trap, time of flight (TOF), orbitrap, and Fourier transform (FT) or Fourier transform ion cyclotron resonance (FT-ICR) [38,71]. Various plant lipidomics studies utilising different separation, and ionisation methods, and mass analysers are listed in Table 1. 

**Figure 2:**
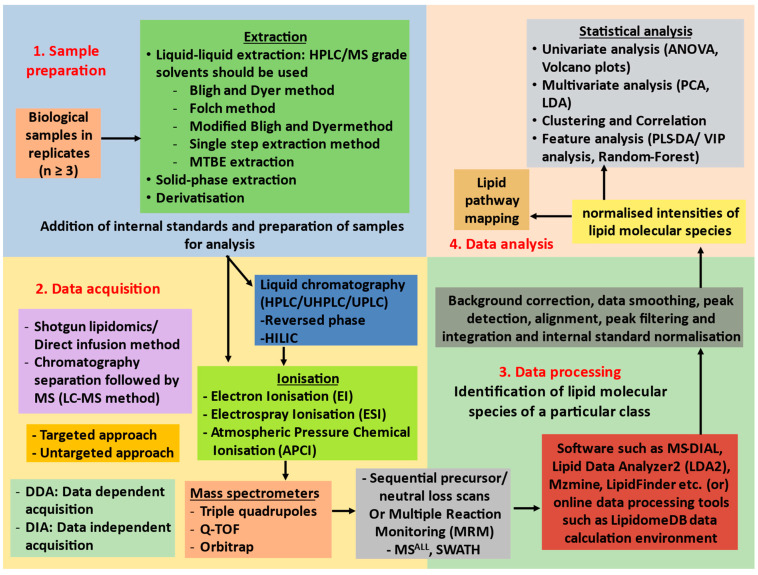
Flowchart of quantitative mass spectrometric analysis of lipid molecular species. Abbreviations: MTBE, Methyl tert-butyl ether; HPLC, High-Pressure Liquid Chromatography; UHPLC, Ultra-High-Pressure Liquid Chromatography; UPLC, Ultra-Pressure Liquid Chromatography; HILIC, Hydrophilic Interaction Chromatography; Q-TOF, quadrupole-Time of Flight; MS^ALL^, All ion fragmentation; SWATH, Sequential Window Acquisition of all Theoretical fragment-ion spectra; ANOVA, Analysis of Variance; PCA, Principal Component Analysis; LDA, Linear Discriminate Analysis; PLS-DA, Partial Least-Square Discriminate Analysis.

MS-based systems can be classified into two major analytical platforms: direct infusion MS (ESI-MS) and liquid chromatography coupled MS (LC-ESI-MS) [72,73]. The direct infusion of MS, also called shotgun lipidomics, is performed at a constant concentration of lipid solution without any previous separation of lipids before entering the ionization step of MS. In contrast, the LC-MS involves the separation of lipid classes/species, before ionisation, in an LC column, with the concentrations of lipid species varying with specific elution time [66]. The separation systems include the reverse phase (RP) or hydrophilic interaction chromatography (HILIC). The separation of lipids in an LC column depends on their specific physicochemical properties such as polar head group, acyl chain length, and number of double bonds. Therefore, the LC-MS provides better separation efficiency, high sensitivity, and specificity. Despite its relatively lower sensitivity than LC-MS, shotgun lipidomics has gained popularity in recent times due to its ability to quantify numerous lipid species accurately in a relatively short runtime and its suitability for large-scale analysis [23,73,74,75]. For direct infusion, the sample is loaded into a syringe pump from which the sample is continuously infused into an ion source. An ESI-QqQ MS system in MRM (multiple-reaction monitoring) mode is a well-adapted setup for targeted detection and quantification of lipids [5,76]. Summarised representation of lipidomics workflow is shown in Figure 2. 

Plant lipidomics mainly utilises ESI-MS/MS (triple quadrupole tandem MS) to analyse and collect considerable mass spectral data. Each spectrum is specific for a particular lipid molecular species; having a joint head group as lipids in a class will produce common head-group fragments in the MS. A lipid profile consists of a series of head-group-specific scans. Scanning the spectrum in different MS modes allows using the fragments originating from the head group as the criteria for detection [90,91,92]. Lipid molecular species will be identified as a head group and mass, which can be correlated with acyl carbon numbers of one or more acyl chains and several double bonds [5,23,74].

The well-characterised response of plants to cope with high-temperature stress is by changing the membrane lipid composition [63]. Data about such complex lipid alterations can be recorded with high-throughput mass-spectrometry-based analytical platforms. Several studies have employed MS-based lipidomics to profile changes in plant membrane lipids [4,5,7,8,9,10]. The ultimate goal of lipidomics is to determine the relative or absolute abundance of one, several, or all lipid species present in the sample [66,67]. Relative quantification measures the pattern change of lipids between treatments. This approach is helpful for biomarker discovery and for characterizing the response of treatments. Absolute quantification determines the mass levels of individual lipid species and derives the total amounts of lipid classes and subclasses in a sample [66]. For absolute quantification, either an external or internal standard must be used to quantify the analyte of interest in MS analysis [93]. A stable isotope labelled internal standard per lipid species is preferred for quantification, but obtaining such a labelled standard for each lipid species is not practical. Therefore, commercially available non-native (non-physiological) lipid internal standards are commonly used, i.e., the internal standard should be absent from the sample or present at extremely low abundance, such as those with odd- or short-chain fatty acids [94]. The basic principle behind quantification of a lipid species with the internal standard is based on ratiometric comparison:

*I_u_/I_s_* = (*A_u_/A_s_*) × (*C_u_/C_s_*) (1)

where *I_u_* and *I_s_* are the respectively measured ion intensities of the analyte and internal standard from a baseline-corrected MS spectrum; *C_u_* and *C_s_* are the unknown concentration of the analyte and the known concentration of the internal standard, respectively; *A_u_* and *A_s_* are the response factors of the analyte and internal standard(s), respectively, under identical experimental conditions [95,96].

## 5. Plant Membranes and Lipid Remodelling under High Temperatures

Plants, as sessile organisms, are exposed to many stresses. The plasma membrane acts as the cell insides and external environment interface. Plant cell membranes are made up of complex assemblies of lipids and proteins that together are destined to perform diverse functions in the cell, such as compartmentalisation of cell organelles, defining permeability barriers that separate cells from their surrounding environments, and preventing the diffusion of organelle contents [97,98]. Being the interface, membranes ought to maintain stability, structure, and integrity for proper cellular homeostasis [99]. Plant membranes are subjected to alterations and reprogramming in response to environmental conditions. Membranes are the significant targets of most stresses, and stress-induced membrane damage leads to electrolyte leakage, indicating characteristic disruption of cellular homeostasis [100,101]. Hence, maintaining membrane stability is a crucial factor for any kind of stress tolerance in plant [3], and cell membrane thermostability can be considered a criterion to screen tolerant plants [102]. Biological membranes are primarily fluidic bilayers with few patches/regions that can transit from fluidic to gel or bilayer to non-bilayer phases under certain circumstances [103,104]. In bilayer phases (fluid and gel), the polar head groups of lipids are arranged facing the aqueous outer environment on both sides of the membranes, and the non-polar fatty acid chains are packed within the bilayer facing opposite to each other (head group facing outside and tails facing inside) [105]. The *cis-double* bonds in the tail fatty acids introduce kinks into the lipids and are therefore responsible for maintaining optimal fluidity of the membranes and preventing dense packing of lipids [7,77]. In the gel phase, lipids are closely packed, and the fatty acid chains are extended and ordered for dense packing. In contrast to fluidic and gel phases, hexagonal phases are inverted, with polar head groups facing inside and fatty acid tails facing outside [106,107,108]. The gel and hexagonal phases are only minor components in normally functioning membranes under normal conditions. Under stresses such as high temperatures, the number of these minor phase components increases in amount, causing the membranes to lose stability and function. High temperatures cause phase transitions from gel to fluid and fluid to hexagonal phase, leading to ion leakage and consequent cell death [14,109]. HT stress also shoots up the levels of reactive oxidants in the cell. Reactive oxygen species (ROS) then induce peroxidation of membrane polyunsaturated fatty acids, leading to membrane damage, electrolyte leakage, and cell death [110,111,112]. Membrane glycerolipids are oxidised to oxylipin-containing glycerolipids, and trienoic fatty acids are peroxidised, yielding other cytotoxic molecules [113], having deleterious effects on photosynthetic proteins through cleavage, oxidative modifications and irreversible aggregation [111,114].

Aside from plasma membrane damage and electrolyte leakage, photosynthesis is severely affected because the thylakoid membrane is extremely susceptible to high temperatures [115]. Primary light reactions of photosynthesis take place in thylakoid membranes where complexes containing proteins, photosynthetic pigments, and cofactors required for capturing photons, electron transfer, and energy exchange are embedded within the bilayer formed by a peculiar combo of glycerolipids [116,117,118]. Thus, high-temperature-induced thylakoid damage affects the light reactions required to make NADPH and ATP, which are further necessary for reducing CO_2_ to carbohydrates via photosynthetic dark reactions [119,120]. The effect of high-temperature stress on photosynthesis has been studied in various crops such as wheat (*Triticum aestivum* L.) [121,122], rice (*Oryza sativa* L.) [123], sorghum (*Sorghum bicolor* L. Moench) [124], soybean (*Glycine max* L.) [125], maize (*Zea mays* L.) [126], cotton (*Gossypium hirsutum* L.) [127], etc. High-temperature stress studies presented a reduction in chlorophyll pigments, net photosynthesis (Pn), F_v_/F_m_ ratio, electron transport rate (ETR), photochemical quenching (qP), stomatal conductance, and yield. A contrasting increase in non-photochemical quenching (NPQ) represents a disrupted electron flow in the thylakoid membrane [79], sparing more electrons for the generation of ROS. A decrease in photosynthetic rate and subsequent yield loss in wheat under high-temperature stress is found to be due to lipid desaturation, oxidation, acylation, and damage to organelles [8].

Plants have evolved various biochemical and physiological strategies to respond to and adapt to changing environmental conditions. As already mentioned, galactolipids such as MGDG and DGDG (80%) predominate in the thylakoid membrane, the composition and state of the lipid profile of the membranes are essential to maintaining appropriate fluidity and flexibility of the membrane [63,128], which explains its relevance to the intriguing study of lipids in maintaining complex cellular functions such as photosynthesis. To optimise photosynthesis and other cellular processes, plants often adjust the properties of their cellular membranes, such as membrane fluidity, lipid composition, and fatty acid saturation levels, in response to temperature stress [7,8,74,77,129]. Alterations in membrane lipids to adapt to adverse conditions can be referred to as remodelling. Lipid remodelling refers to decreasing or increasing specific lipids [7,13]. Such remodelling responses help prevent the increasing degree of phase transition of membranes (bilayer to non-bilayer) and avoid their damage at high temperatures [130].

**Figure 3:**
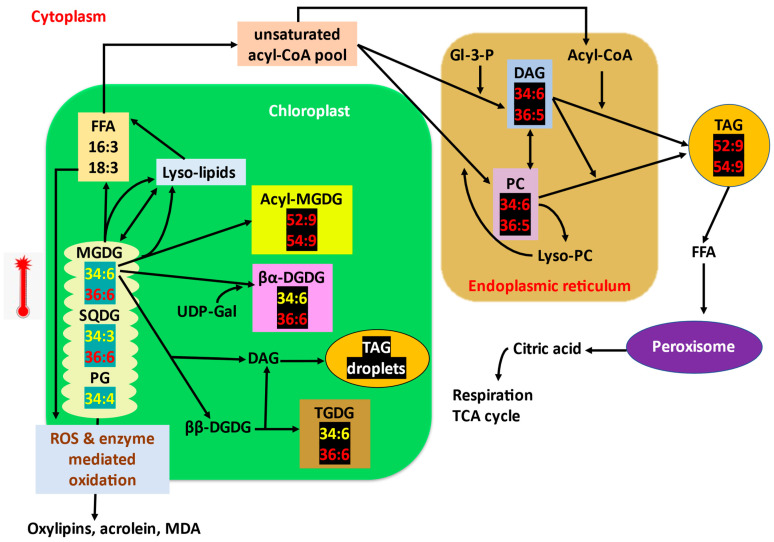
Overview of the trienoic fatty acid containing plastidic lipid remodelling strategies under high temperatures. Levels of non-bilayer forming MGDG are reduced and directed towards acyl-MGDG, DGDG, PC, TAG, TGDG, and oxylipins. Trienoic fatty acids from plastidic lipids are removed and transiently stored in TAG as lipid droplets. The figure showing the remodelling steps in reducing the unsaturated fatty acids from chloroplast lipids is modified from Higashi and Saito [63]. Abbreviations: FFA, free fatty acid; CoA—coenzyme A; 16:3, hexadecatrienoic acid; Gl-3-P, glyceraldehyde-3-phosphate; MGDG, monogalactosyl diacylglycerol; DGDG, digalactosyl diacylglycerol; SQDG, sulfoquinovosyl diacylglycerol; TGDG, trigalactosyl diacylglycerol; PG, phosphatidyl glycerol; PC, phosphatidyl choline; TAG, triacyl glycerol; MDA, malondialdehyde.

Thus, specific membrane lipid profile changes or alterations can be associated with high-temperature tolerance and susceptibility [14,55]. Lipid composition and fatty acid saturation are the significant factors responsible for a membrane’s performance under temperature stress [44]. Readjustment of various glycerolipid biosynthetic pathways under stress conditions might be helpful for the plants to effectively remodel the membrane property to tolerate high-temperature stress [45,81]. Several of such membrane lipid alterations under stress conditions have been summarised in Figure 1 and Figure 3.

- Double bonds in plant membrane lipids exist in *cis* configuration, which creates a bend in the fatty acid structure and avoids dense packing of lipids [7]. During high-temperature stress, this bend/kink in fatty acids makes the membranes hyper-fluidic and destabilises the membrane structure. A decrease in degree of unsaturation of membrane glycerolipids is a well-known response to encounter high-temperature stress. Polyunsaturated (18:3/16:3) fatty acids content is decreased, whereas the content of less unsaturated (18:2/18:1/16:1) and saturated (16:0) fatty acids increase. Levels of 18:2 containing glycolipids such as 34:2-DGDG, 36:4-MGDG/DGDG/SQDG, 36:5-MGDG/DGDG/SQDG are increased (lipids highlighted in black in Figure 1, in prokaryotic pathway), whereas levels of trienoic fatty acid lipids such as 34:6-MGDG, 34:3-SQDG, 36:6-MGDG/SQDG, 34:4-PG are decreased (lipids highlighted in blue in Figure 1, in prokaryotic pathway). Increasing the levels of saturated and monounsaturated fatty acids provide tolerance to high-temperature stress by reducing the membrane fluidity, which is increased as impact of high temperatures [7,8,39].
- The decrease in unsaturation of lipids is predicted to be a coordinated mechanism of lipid turnover and intracellular lipid trafficking [4]. The trienoic fatty acids responsible for the fluidity of membranes are released from the membrane and stored as transient TAGs (lipid droplets) within chloroplast or cytoplasm [6,131,132]. The expression of several fatty acid desaturases responsible for inserting double bonds in fatty acids is decreased, and the saturated lipid species such as 16:0/18:1/18:2 are synthesised without further desaturation and inserted into the membranes [4,9,10]. In other words, unsaturation levels of membrane lipid species decreased, whereas that of synthesised TAGs increased (TAGs 52:9 and 54:9 increase; Figure 3).
- High-temperature stress decreased the levels of 16:3-containing plastidic lipids by producing triacylglycerols and stored them as transient oil bodies within the cell. Unsaturated fatty acids are released from the membrane lipids and are transferred to DAGs by an acyltransferase (PDAT1) to form TAG [133] as a transient mechanism to store free fatty acids prior to lipid recycling or degradation pathway as the β-oxidation pathway for fatty acid turnover is reported to be slow [4,132,134]. Highly unsaturated pool of PC and DAG (34:6, 36:5) (direct precursors for TAG) is formed as by-product of membrane lipid remodelling and is later converted to TAGs [135,136] (Figure 3). *pdat1* mutant *Arabidopsis* is highly sensitive to high temperatures as it cannot accumulate TAGs.
- Saturation and desaturation of fatty acids occurs in a course of several enzymatic reactions involving oxidation-reduction reactions that require more energy to do so and would become a poor trade for plants encountering stress. Zheng et al. [12] hypothesised and confirmed an alternative lipid remodelling strategy that plants employ to cope with the frequent temperature fluctuations. Snow lotus (*Saussurea medusa*) in Alpine scree preserved its membrane functions by maintaining the same degree of membrane lipid unsaturation, but instead varies the membrane lipid composition through some less energy-demanding mechanisms such as head group turnover or glycerolipid pathway balancing [44]. The head group exchange reactions are rapid, and the energy required is less than that required for saturation–desaturation reactions and these exchange reactions are important processes in lipid metabolisms [13,137].
- Rather than reducing the double bonds of already-made membrane fatty acids, plants alter the lipid biosynthetic pathways to sequester less-saturated lipids into membranes, i.e., high-temperature stress increases the eukaryotic pathway contribution for galactolipids synthesis [63]. The proportion of DAGs derived from eukaryotic and prokaryotic pathways for further synthesis of other glycerolipids is altered under high-temperature stress. Eukaryotic-pathway-derived DAGs are less unsaturated (containing 18:2 fatty acid) than those derived from the prokaryotic pathway [138]. High-temperature-stressed wheat plants were found to channel more saturated eukaryotic DAG species from ER to chloroplasts than more unsaturated prokaryotic DAGs to maintain the fluidity and stability of thylakoid membranes [44]. Similar results were marked by Zoong Lwe et al. [10] with high-temperature-stressed *Brassica carrinata*.
- The proportion of bilayer-forming (DGDG, SQDG, PC, PG) and non-bilayer-forming lipids (MGDG, PE) is also a major factor determining the stability of membranes [139]. Since MGDG is the only non-bilayer-forming lipid in thylakoid, it is considered to be crucial for the formation and proper stacking of grana, and also is an integral component of reaction centres [52,140]. Plants increase the DGDG to MGDG ratio to improve thylakoid stability and thermotolerance [141]. High-temperature stress tolerance is attributed to the increased level of a bilayer-forming lipid, in this case, DGDG, facilitating the stability of the membrane. Drought-primed tall fescue plants maintained a higher ratio of DGDG/MGDG and maintained optimum fluidity and stability of thylakoids under subsequent high-temperature exposure [84]. DGDG/MGDG ratio is a factor for drought tolerance in maize, where a tolerant cultivar has a high ratio compared to susceptible cultivars [89]. DGDG synthase activity is increased, which converts MGDG to DGDG. The level of MGDGs is also decreased by the action of specific lipases [17,63] (Figure 3).
- Several catabolic processes can also contribute to galactolipid remodelling under high-temperature stress. Lipases such as HIGH-TEMPERATURE INDUCIBLE LIPASE 1 (HIL1) are found to be involved in chloroplast glycerolipid remodelling under high-temperature stress by cleaving 18:3 fatty acids from glycerolipids, especially MGDG [17].
- High-temperature stress increases the levels of oxidised lipids (ox-lipids) such as ox-PCs, ox-Pes, and ox-MGDGs in wheat leaves [7,8]. Membranes serve as both the source of ROS and reservoir to dump ROS [142,143,144]. Unsaturated fatty acids in membranes are oxidised enzymatically by chloroplast lipoxygenases (LOXs) or non-enzymatically by direct action of ROS (Figure 3). Thus, membrane lipids take up the ROS and prevent their damaging effects elsewhere in the cell [145]. The non-enzymatic oxidation of trienoic fatty acids such as 18:3, acts as an immediate mechanism in consuming the ROS produced during stress without activating the antioxidant molecular responses. The ox-lipids, such as OPDA (oxo-phyto dienoic acid), thus produced will then be subjected to β-oxidation to produce jasmonic acid [146], which can provide tolerance to various stress [147]. The high-temperature-tolerant wheat genotype retained relatively lower levels of oxylipins and MDA (end product of lipid peroxidation) than the susceptible genotype. Thus, the level of ox-lipids can be considered as a biomarker for high-temperature tolerance or susceptibility of plants [7,11,14].
- Extra-plastidial lipids such as PC, PE, PS, and PI are also remodelled. Unsaturated phospholipid contents are decreased (34:2-PC/PE/PI, 36:4-PC/PE, 36:5-PC, 36:6-PC) (lipids highlighted with blue in Figure 1, eukaryotic pathway), while the levels of saturated and monounsaturated fatty acid containing phospholipids are increased (lipids highlighted with black in Figure 1, eukaryotic pathway) [4,7,148]. Under high-temperature extremes, levels of lyso-phospholipids increase sharply, indicating the removal of fatty acid from membrane lipids. Thus, lyso-phospholipid content can be considered a sensitive indicator for plant stress response [5].
- Sterol lipid or sterol glycoside contents were also found to be increased under high-temperature stress [7]. Phytosterols stabilise membranes and promote ordering of structural membrane components. SGs help to eliminate membrane phase transitions from bilayer to non-bilayer phases at high temperatures [149].
- High-temperature stress induces acylation of the galactose moiety of MGDG, yielding acyl-MGDG such as 52:9/54:9-acyl-MGDG and lyso-MGDG [136,150]. Increased acyl-MGDG indicates damaged chloroplasts [45]. Lyso-lipids can be again reacylated to MGDGs or can be hydrolysed to yield fatty acids for TAG synthesis. (Figure 3).
- Acylated and oxidised lipid levels rise concomitantly during stress and can be used for screening genotypes for stress tolerance [45].
- *Arabidopsis* leaves accumulated higher levels of 34:6/36:6-TGDG under high-temperature stress. The level of TGDG is high in high-temperature-stressed *pdat1 Arabidopsis* mutant, in which TAG synthesis to trap free trienoic fatty acids is impaired. This provides that the consumption of MGDG to make TGDG via galactosylation (Figure 3) contributes to lipid remodelling under high-temperature stress [63,136].

## 6. LIPIDOTYPE: Lipids and Lipid Remodelling as Potential Biomarkers for High-Temperature Tolerance

High-temperature stress affects most physiological processes of plants, of which all mechanisms of photosynthesis are highly susceptible to high temperatures as the photosystem II, ATP-generating system, and carbon fixation by Rubisco are adversely damaged [151,152]. A reduction in chlorophyll content, swelling, and damage of the thylakoid membrane leads to malfunctioning PSII and associated electron transport chain, producing ATP and NADPH required for CO_2_ fixation [153]. Disrupted electron transport eventually reduces photosynthetic efficiency and spares more electrons for the generation of ROS, which later has its own range of aftermaths [154]. Chlorophyll fluorescence measurements can be used to probe the status of PSII and linear electron transport rate towards various stresses [155,156,157]. Fluorescence induction parameters such as maximum quantum yield of PSII (F_v_/F_m_), effective quantum yield of PSII (ϕPSII), maximum variable fluorescence (F_m_/F_o_), the efficiency of water splitting complex on donor side of PSII (F_v_/F_o_) and quenching parameters can detect different levels of stress-induced photosynthetic damage [158]. Low values of F_v_/F_m_, ϕPSII, photochemical quenching (qP), and electron transport rate (ETR) indicate low photosynthetic efficiency [159,160]. The altered chlorophyll fluorescence kinetics and decreased photosynthetic rate under high-temperature stress can be attributed to the reduced concentration of photosynthetic pigments, membrane lipid alterations, and organelle damages [7,8,161]. It was found that the decrease in photosynthetic rate in wheat was found to be due to an interplay between thylakoid membrane damage, membrane lipid changes, and damage to cell organelles. The lipid alterations under high-temperature stress indicate an increase in activities of oxidising, glycosylating, and acylating enzymes and decreased activity of desaturating enzymes [7,8,79]. 

**Figure 4:**
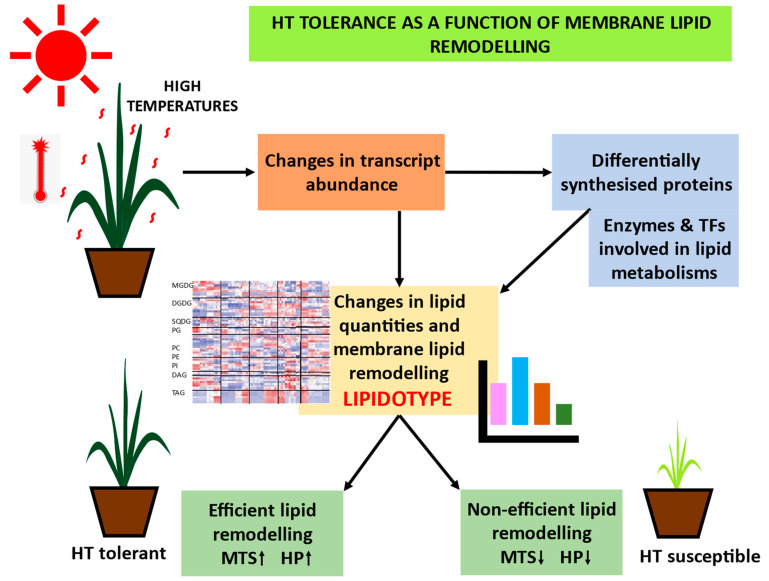
Representation of the hypothesis of considering high-temperature stress tolerance as a function of membrane lipid remodelling. Several lipids and lipid alterations can be considered intermediate phenotype for HT stress tolerance/susceptibility to select tolerant genotypes. Abbreviations: HT—high-temperature; TFs—transcription factors; MTS—membrane thermostability; HP—high-temperature stable photosynthesis.

Lipids and their metabolic products and derivates can act as a physical barrier or as a signalling molecule that directs plants to adapt to various biotic and abiotic stressors [21,162,163]. As discussed earlier in Section 5, the physical and physiological properties of membranes are affected by various environmental factors, and plants alter several metabolisms to adapt to adverse conditions. Membrane lipid remodelling is one of the strategies plants employ to combat abiotic stresses, including temperature stress. This lipid remodelling, which is responsible for maintaining the integrity of membranes under stress conditions, is genotype-specific, i.e., remodelling strategies and the extent or efficiency of remodelling vary with genotypes (as shown in Figure 4) [4,7,9,10,14,55,78,86]. One such well-characterised adaptive remodelling in plants is the decrease in double bond numbers of membrane lipids at high temperatures. Other alterations besides those discussed in Section 5 may also contribute to tolerance, warranting further lipidomic studies across different crops and genotypes.

Narayanan et al. [6] showed the differential lipid remodelling responses of two wheat varieties, with Ventnor (high-temperature-tolerant) showing more efficient remodelling under high temperatures than Karl 92 (high-temperature-susceptible). Both genotypes underwent lipid remodelling to preserve the membrane architecture and functions. The genotypes differed in their ability to accumulate sterol derivatives such as sterol glycosides (SGs) and saturated acylsterol glycosides (ASGs), with Ventnor accumulating higher amounts of SGs and saturated ASGs compared to Karl 92. The high-temperature stress study by Narayanan et al. [9] on two soybean genotypes showed that the tolerant DS25-1 genotype maintained less relative membrane injury compared to the susceptible genotype DT97-4290. The high-temperature tolerance of DS25-1, attributed to the stable cell membrane, is the result of efficient membrane lipid remodelling, as the trait (cell membrane thermostability) is closely related to membrane lipid composition. High-temperature stress studies on wheat by Narayanan et al. [7] and Djanaguiraman et al. [8] showed the formation of ox-lipids by either direct oxidation or mediated by lipoxygenases. The level of ox-lipids may indicate the chemical status of the plant and the plant’s innate tolerance level to high-temperature stress, since the membrane unsaturated fatty acids may serve as a dump for the reactive oxidants. Xu et al. [129] examined the membrane lipid remodelling of two wheat genotypes with contrasting response to hypoxia stress. The tolerant genotype CIGM90.863 showed efficient lipid remodelling mechanisms to maintain the membrane bilayer structure when compared to the susceptible genotype Seri M82. 

Various lipid biosynthetic pathways undergo changes, and the relative contribution of the two lipid biosynthetic pathways is also changed. Several enzymes or genes involved in the pathways are subjected to differential expression under high-temperature stress. Higashi et al. [3] published the lipid changes during high-temperature stress and the associated differential expression of genes involved in the fatty acid synthesis, lipid biosynthesis, and β-oxidation cycle. Such lipid metabolism gene expressions also vary between genotypes, which show differential response to high temperatures. Expression pattern of the genes *FAD3A* and *FAD3B,* responsible for fatty acid desaturation, varied between the two soybean genotypes [9]. The varied gene expression also correlates with the varying lipid data, suggesting the role of reduced *FAD3* expression to be a part of the membrane lipid remodelling strategy in maintaining the membrane stability under high-temperature stress and of a potential target for breeding [9,10,55]. 

Thus, such lipid alterations can be considered a phenotype for high-temperature-stress tolerance or susceptibility and can act as a potential biomarker for selecting HT-tolerant genotypes (Figure 4) [9,55]. Although such remodelling mechanisms have already been studied in plant species such as *Arabidopsis* [6,8], wheat [7,8,11,79], soybean [9], Brassica (*Brassica juncea* L.) [10], and peanut (*Arachis hypogaea* L.) [78], limitations still exist regarding the lipid remodelling responses of crops under various stress conditions. Therefore, further studies are required to determine the relationship between lipid remodelling and high-temperature stress tolerance in different crops. Determination of such a particular lipid alteration or a specific lipid biomarker responsible for high-temperature stress tolerance would aid in the screening of genotypes for the same and in determining the genetic variation responsible for the biomarker trait. The specific LIPIDOTYPE (lipid metabolite or the lipid alteration(s)) contributing to stress tolerance may be controlled by one or a few genes. Further research utilising multiple integrated omics strategies on various species and genotypes with differential response to high-temperature stress to pinpoint the lipidotype variability could and will help in identifying the genes and genetic factor responsible for the variation in the trait and could potentially be used in breeding programs.

## 7. Bridging the Gap: Genotype–LIPDOTYPE–Phenotype: Lipidomics-Assisted Breeding (lGWAS) for High-Temperature Tolerance

Plant species differ widely in their high-temperature stress response from being tolerant to susceptible. High-temperature stress tolerance is a polygenic trait; genotypic variability of HS response was found to be controlled by many genes, generally with a minor cumulative effect over the phenotype [164,165]. In other words, the high-temperature stress response of genotypes is quantitative in nature; it is governed by several small effects or epistatic QTLs, and this is expected to be so as the effect of high-temperature spans over a wide range of cellular processes [166,167,168]. Hence, breeding for high-yielding, high-temperature-tolerant crops is challenging as the genetic inheritance of high-temperature tolerance is still poorly understood, and validated QTLs/cloned genes for high-temperature tolerance are scarce [169]. Furthermore, the influence of the trait through genotype–environment interactions exacerbate the process. Therefore, the need arises for the identification of large-effect QTLs and to develop associated markers that can be used for marker-assisted breeding [55].

Employing traditional breeding strategies such as direct selection for yield under stress conditions to develop HS-tolerant crops may have some limitations due to the low heritability and control of the trait by a complex network of major and minor QTLs [168,170,171]. The complexity of the high-temperature tolerance trait, the lack of high-throughput phenotyping for the selection of tolerant genotypes, and the critical effect of genotype–environment (GxE) interactions over the trait stand as major bottlenecks for breeding HT crops [167,172]. Numerous studies have been conducted on different crops to identify associations for HS tolerance. However, the effect of genetic associations on such complex traits is often small, and information about the biological processes underlying such traits is lacking in most crops [173,174]. Therefore, targeting intermediate traits that are closely related to the physiological and biochemical status of the stressed plants could reduce the complexity of HT trait [175,176]. Physiological trait-based breeding has been proven to be an ideal strategy for transferring QTLs/gene(s) conferring high-temperature tolerance [169,177,178]. Physiological traits such as photosynthetic efficiency, lower respiration rates, membrane thermostability [9,179,180], canopy structure, delayed senescence, reproductive traits, and harvest index can be considered. Desirable characters for a high-temperature-tolerant variety include higher photosynthetic rates, enhanced thermostability of membranes [9,181,182], low ROS level or increased antioxidant potential [115,183], and stable seed set/grain production under high temperatures [166,184]. 

The metabolome is defined as the last receiver of genetic information flow to attain the desired phenotype; in other words, metabolites are considered to be the bridge between genotypes and phenotypes [175,185,186,187]. A metabolic trait can be either a functional intermediate or a correlated biomarker for the physiological status of a plant [174,176,188]. Plant metabolic networks are very complicated and consist of many interconnected biochemical pathways that are affected by different environments and stress conditions. The expression of genes is changed by various stress conditions, such as high temperatures, that can alter the qualitative and quantitative status of metabolites, making the study of metabolites arduous [189,190]. High-temperature stress suppresses plant growth, development, and reproduction by disrupting major cellular processes such as photosynthesis, primary and secondary metabolisms, lipid and hormonal signalling and source–sink relations, and ultimately leads to yield penalties [189,190,191]. The predominant effect of HS is on the cellular membranes, particularly thylakoid membranes, where HS causes grana destacking and concomitant impaired photosynthetic light reactions [115,192]. The importance of membrane properties and the membrane lipid remodelling strategies to combat high-temperature stress was discussed in Section 5, and so HS tolerance can be considered as a function of membrane lipid remodelling [14,55,193]. An example of one potential method to improve high-temperature tolerance is demonstrated by a tobacco mutant whose chloroplast FAD gene has been silenced, which exhibits excessive high-temperature tolerance by maintaining relatively low fatty acid unsaturation levels [15]. Characterizing lipid remodelling mechanisms is quite challenging because of the large number of intermediates, interactions between subcellular compartmentalised distinct lipid pathways, and by the structural and combinatorial diversity of the lipids itself [194,195]. The study of plant lipids and plant membrane lipidomics is gaining importance as the high-temperature stress alters the membrane properties, and plants respond to high-temperature stress by lipid remodelling to maintain membrane stability. The degree of such lipid alterations varies according to the genotypes. Genotype-specific lipid remodelling under various stress conditions has been studied in some crops such as wheat, rice, soybean, grapevine (*Vitis vinifera* L.) [7,89,130]. Irrespective of complex phenotypes under field conditions, one can easily profile relative contents of the metabolome, which may be directly or indirectly related to the interested phenotype [196] and could possibly bridge the gap between genotype and phenotype [185]. Therefore, the identification of functional candidate genes or the association underlying the quantitative variation in high-temperature stress-induced lipid remodelling may be the potential target that can be manoeuvred for breeding high-temperature-tolerant crops.

Homologous-sequence-alignment-based reverse genetic strategy would be a straightforward method for identification of metabolite/lipid candidate genes [175]. One such example is that of wheat benzoxazinoid genes cloned against their maize orthologues via this strategy [197]. Similarly, Shavit et al. [198] identified the dioxygenase gene *BX6* in tetraploid and diploid wheat by homologous sequence comparisons and phylogenetic analysis. However, this procedure may not work in all instances when the metabolic genes have less sequence homology to the identified orthologs. In such a case, a sequence-alignment-based procedure might cause extensive labour. In polyploid crops such as wheat, the chance of identifying the targeted metabolite genes becomes lower and the work becomes tedious and laborious [175,199]. Co-expression-analysis-based gene identification has been used to identify lipid metabolism genes and genes involved in oil biosynthesis [200,201,202]. Co-expression analysis can enhance the predictive power of detecting functional gene homologs [203,204,205,206]. However, this strategy depends on existing knowledge of gene functions and requires gold-standard experimentally validated predicted function of genes, and this even remains partial for the model plant *Arabidopsis* [194,206]. Therefore, need arises for considering additional omics datasets, such as metabolomics and lipidomics, to identify candidate genes [188,194,207,208,209]. Another way to identify a metabolic gene is to employ an integrated omics approach combining metabolomics with forward and reverse genetics [175,189,210]. This approach has been proved to be efficient by recent publications unearthing numerous metabolic quantitative trait loci (mQTLs) hotspots for metabolite classes within species [211,212,213,214,215,216]. The technique utilises quantitative genetic strategies such as quantitative trait loci (QTL) mapping or genome-wide association studies (GWAS), along with large-scale targeted or untargeted metabolomics, and integrates both to obtain valuable insights into the genetic and biochemical basis of the metabolites. The output of these methods, named mQTL and mGWAS, respectively, are the linkages/associations between metabolite quantities and chromosomal locations in the mapping population or the diversity panel utilised [217,218,219,220]. The resulting associations should be further evaluated for the potential candidate genes underlying the metabolic variation. As such, markers can also be developed for the identified linkages and can directly be utilised in maker-assisted breeding for developing desired crops [175,176]. The mGWAS approach has advantages over mQTL, as the GWAS mapping utilises the vast number of diverse accessions/populations and relies on natural linkage disequilibrium progressively generated by countless ancestral recombination events [211,221], whereas QTL mapping populations derived from two or fewer parental genotypes are not scalable to investigate the qualitative and quantitative diversity of the metabolites as it is limited to the few recombination events [222,223]. Several mGWAS studies has been executed in major crops such as tomato (*Lycopersicon esculatum* L.) [216,224], rice [212], maize [211], wheat [225], barley [215], and foxtail millet (*Setaria italica* L.) [220]. 

**Figure 5:**
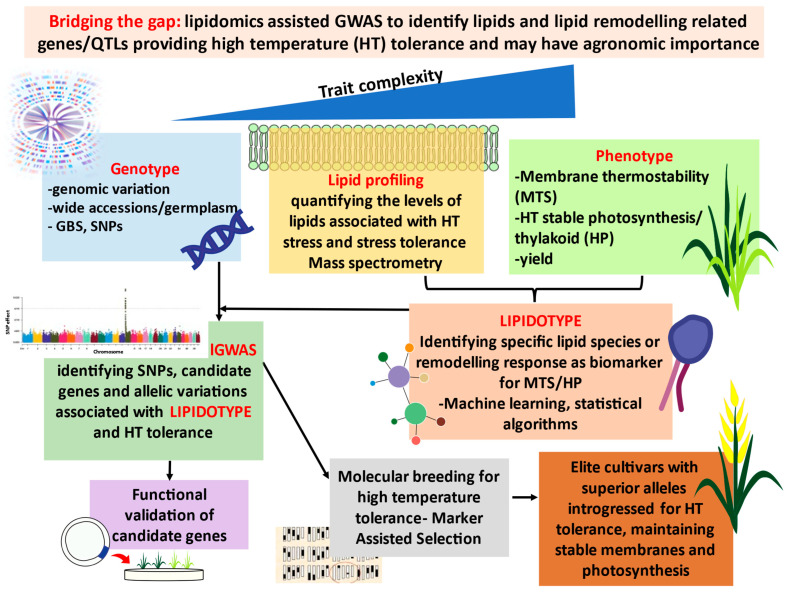
Bridging the gap: Lipidomics-assisted GWAS (lGWAS). Experimental strategy of the proposed lGWAS workflow, providing a novel route for identifying lipids and lipid remodelling-related QTLs/genes providing high-temperature (HT) tolerance and may have agronomic importance. Abbreviations: GBS—Genotyping-by-sequencing; SNP—Single-Nucleotide Polymorphism.

As this approach is still young, only few reports have been registered on the identification of abiotic-stress-tolerance-correlated mQTLs [176,215,226] but demonstrate potential utilisation in physiological trait-based breeding for developing stress-resilient crops. As discussed, high-temperature tolerance is a complex trait, and the thermostability of membranes such as thylakoid (measured by electrolyte leakage or relative cell membrane injury) and photosynthetic responses (chlorophyll fluorescence measurements) can be considered physiological traits for selecting high-temperature-tolerant crops [9,167,168,227]. Given that membrane thermostability (MTS) and high-temperature stable photosynthesis (HP) are also complex traits, and the genetic basis of such traits remain unclear, a promising strategy would be to investigate the genetic basis of a less complex intermediate phenotype and then link back with the complex phenotype of interest (MTS-HP) (Figure 4) [176,211,228]. Since the above-stated traits are related to the lipid composition of membranes [8,9,14,55], LIPIDOTYPE (lipid molecules or specific lipid remodelling response) can be used as biomarker for high-temperature-tolerance-associated MTS/HP via. the route genotype–MTS/HP-LIPIDOTYPE (L)–phenotype (HT). In brief, the proposed strategy would be achieved by (as shown in Figure 5) (1) using a diverse population to capture the maximum variability in alleles (GWAS), (2) genotyping with dense markers such as SNP (Single-Nucleotide Polymorphism), or usually genotyping-by-sequencing or by resequencing, (3) electrolyte-leakage-based assay of membrane thermostability (MTS)/high-throughput phenotyping-assisted measurement of photosynthetic parameters such as thylakoid damage (F_0_/F_m_), ϕPSII, qP, NPQ, Pn (HP) under high-temperature conditions, (4) LC-MS-based high-throughput untargeted lipidomics approach to obtain data about the membrane lipids and their alterations, (5) identifying and characterising the particular lipidotype (L) biomarker for targeting and mining of genetic determinants underlying the natural variation of lipidotype (L) and MTS-HP through big data mining and genotype–phenotype predictions, (6) identifying the lipidotype QTLs (lQTLs) associated with high-temperature-tolerance phenotypes MTS-HP, (7) identification of genes and its functional validation, and (8) development or markers to be used in marker-assisted breeding for selection of high-temperature-tolerant crops.

## 8. Conclusions

With developments and advances in genome sequencing, large-scale metabolomics, bioinformatics, and machine learning pipelines, candidate gene identification for any metabolite/lipid is now possible. However, knowledge about the HT stress-induced quantitative variation in any lipidotype is still lacking and needs further extensive investigation of the lipid remodelling responses of plants. Utilising a genome-wide association panel for the work can overcome the inherent drawbacks of segregating populations, and when the experiment is designed more comprehensively with powerful statistical methods, it will result in accurate deciphering of the genetic association. 

Another dimension of omics, transcriptomics, can also be clubbed together with GWAS, which would identify statistical association (eQTL) between genomic regions (markers) and the expression level of a particular gene. Possible multi-omics design including genomics, phenomics, lipidomics and transcriptomics can further improve the targeting potential of the experimental procedure, which would ultimately lead to the development of efficient markers for breeding stress-resilient crops. 

## Figures and Tables

**Table 1:** List of comparative lipidomics studies referred along with the method of analysis.

Plant Species	Method Used	Stress Condition	Reference
Creeping bentgrass (*Agrostis stolonifera*)	GC-MS/MS	High temperature	[77]
*Brassica carrinata*	ESI-MS/MS	High temperature	[10]
Peanut (*Arachis hypogea*)	ESI-MS/MS	High temperature	[78]
Soybean (*Glycine max*)	ESI-MS/MS	High temperature	[9]
Wheat (*Triticum aestivum* L.)	ESI-MS/MS	High temperature	[7] [8] [79]
*Arabidopsis thaliana*	LC-ESI-MS/MS TLC, GC, ESI-QTOF LC-ESI-MS/MS ESI-MS/MS ESI-MS/MS ESI-MS/MS	High temperature - High temperature High temperature Freezing stress Low temperature	[4] [80] [17] [6] [5] [81]
*Arabidopsis thaliana*	LC-ESI-MS	-	[82,83]
Tall Fescue (*Festuca arundinacea*)	ESIMS/MS	Drought priming and high temperature	[84]
*Paspalum vaginatum*	GC-MS/MS	Low temperature	[85]
Sorghum (*Sorghum bicolor*)	ESI-MS/MS	Low temperature	[86]
Rice (*Oryza sativa*)	ESI-MS/MS	Low temperature Hydric and high temperature	[81] [39]
*Craterostigma plantagineum* *Lindernia brevidens* *Lindernia subracemosa* *Arabidopsis thaliana*	QTOF-MS	Desiccation tolerance	[87]
Tomato (*Solanum lycopersicum*)	UHPLC-APCI-QTOF-MS	High and low temperatures	[88]
Maize (*Zea mays*)	TLC, GC-FID	Drought	[89]
*Saussurea medusa* *Crucihimalaya himalaica* *Arabidopsis thaliana*	ESI-MS/MS	Fluctuating temperatures	[13]

## Data Availability

Not applicable.
